# Mr. Lowe on a Supposed Vegetable Poison

**Published:** 1812-09

**Authors:** W. Lowe

**Affiliations:** Stockport


*To the Editors of the Medical and Physical Journal.*

GENTLEMEN,

VEGETABLES, containing a poisonous principle, are sometimes accidentally mingled with, and frequently are mistaken for, others, that are usually employed as food for man; and it is only when they are eaten in considerable quantity, so as to produce immediate and violent effects, that their presence is ascertained or suspected. Such, for instance, is the case with some of the deleterious *Fungi*, by being used as mushrooms, cicuta as parsley, &c. Nor have we, I believe, any positive tests, whereby we can clearly distinguish one vegetable poison from another, either by chemical analysis, or by the symptoms they excite; or are we able to demonstrate, after death, that a vegetable poison has produced such event.

My present reference to this subject, is in consequence of having been lately called to witness one of the most afflictive scenes of misery that can be conceived. Some oatmeal had been been purchased at a shop in this town, about ten days prior to the period I am going more particularly to dwell upon; which oatmeal, I understand, produced very alarming effects: but, not having seen the cases, am unable to describe the symptoms.

On Friday evening, the 3d of the present month, a load of meal was purchased at the same shop by the governor of our poor's house, but which was a part of another lot, although both lots came from the neighbourhood of Sheffield. The following morning, breakfast was made of this meal, in an article called thick-pottage, eaten with milk by some, and with treacle by others. It was boiled in a large iron pan, always used for the purpose.

On the Saturday morning, at eight o'clock, about eighty people, comprehending all the intermediate ages betwixt three and seventy, breakfasted on this substance. At about half past nine of the same morning, a messenger came to desire I would go into the house immediately, for, says the woman, "the children are falling down one after another, and my mistress thinks they are poisoned." When in the house, I was taken into the room where there were three or four children affected in an extraordinary manner. A piercing stare of the eyes, expressive of the most acute suffering; every limb violently agitated; quivering of the lips, and pallid countenances. When questioned as to the seat of their pain, they made no reply, but applied their hands to the forehead. I had not been in the room many seconds, before messengers entered it from every apartment of the house, all consternation, exclaiming, with frantic gestures, they are all dying, men, women, and children. I was thus hurried from room to room, to witness the distress of these poor creatures, without having an opportunity of attempting any thing, for some minutes. In less than a quarter of an hour I saw upwards of fifty affected as before mentioned. Soon after this, they were brought in from the different factories of the town, where some of the young paupers are employed. In less than an hour, betwixt seventy and eighty of these sufferers were on the premises. I had them taken to a grass-plot behind the house, and to be furnished with chairs and benches. From the circumstance of contaminated oatmeal having so recently occasioned an alarm, no inquiry, as to the cause of the mischief, was made, every person immediately concluding that the pottage contained the cause. Many of them vomited immediately on being attacked, but it did not appear at all to alleviate their pain. One girl, about six years old, was seized with convulsions, in little more than an hour after breakfast, breakfast, which continued, with little intermission, for many days.

As early as possible, I procured a large earthen pan, and desired them to bring me a quantity of strong vinegar, at the same time sent home for some of the subcarbonate of potass. I poured into the vessel, at the first, about a gallon of the acid, to which I added a quantity of the potass, but not enough to saturate the acid, and desired the by-standers to pour it into them, while effervescing, without being particular as to the quantity. It was necessary to hold the cup to their mouths, as few of them were able to support it. Some of them vomited soon after taking their draught; others, that had been previously sick, after the draught had no return of sickness; a few were not sick at all, yet had all the other symptoms. Most of those who possessed consciousness, were desirous of frequently repeating their medicine, observing that it eased their pain, and took off the trembling, and that they could see better. I think some of the adults drank as much as a quart of it. Many of the children, and some of the adults, recovered in the space of two hours, complaining only of a little dimness of sight, and of weakness in their legs. A few of the old people, who were previously feeble, after the subsidence of the violent symptoms, were much exhausted, and had a little spirit and water given to them. At ten in the evening, only nine of them complained materially.

The first symptoms, in nearly all the cases, were a remarkable wildness in the eyes, every limb violently agitated; in a few cases, convulsive twitchings, palpitation, confusion of sight in all the cases; the pupils, in many, much dilated, pulse small, and tremulous. In two cases, syncope, with much sobbing, and cold extremities. Two young women, of full habit, had great determination to the head, with an apoplectic purple suffusion in the face. Scarcely any of them could articulate distinctly at the commencement; two of the old people had a little faultering in their speech on the following day, but without any uneasiness; they all pointed to the forehead, as being the seat of torturing pain; many complained exceedingly of pain about the stomach, and from thence to the umbilicus, especially the nine which I found so afflicted at ten o'clock in the evening. In these cases, as the bowels had not been moved during the day, the hydrarg. submuriatis with jalap was ordered, in doses proportioned to their ages. In the morning (Sunday) four of them had been affected with the medicine, and were much better; two had had strong convulsions in the night, and are are complaining of great pain at the stomach, are excessively feeble, pupils much dilated, an enema was directed for each, which produced copious evacuations, and great relief. Three of them had not been affected by the medicine, yet were much better than last night; they had the powder repeated, and I heard no more of them. One of the young women had a return of the convulsive fits in the afternoon. Monday morning, except a little stupor, and weakness of sight, (of which they yet nearly all complained,) only two were considered as requiring attention: the young woman had a return of the fits in the night; in short, this and the other before-mentioned person had a frequent repetition of them for five or six of the subsequent days, but are now perfectly recovered.

When the alarm occasioned by the use of the first parcel of meal was prevalent, and which was said to contain arsenic, I had a little of it brought to me for my opinion. I examined it with as much accuracy as was in my power, but could not detect the most minute portion of any mineral substance. Some of it was sent to Dr. Henry, of Manchester, who informed the applicant that he could not discover any mineral substance in it. A little of the meal from the poor's-house I also submitted to the same tests, with the same result. Its appearance, to the naked eye, is nearly similar to other meal; but, when examined by the microscope, and compared with another specimen, it was found to contain a much less proportion of the white powder, or farina.

The acetous acid has been considered, by some practitioners, as possessing a power of somewhat counteracting the effects of vegetable poisons. From my own experience I am incompetent either to support or to reject that opinion; or, admitting it to possess such power, am I able to shew through what medium, in the animal system, that power is exerted. However, on the supposition of the mischief having been produced by a vegetable poison, I had recourse to the use of it; and, to give it the appearance of medicine, for the purpose of inspiring more confidence in the patients, combined with it the subcarbonate of potass, but in quantity considerably under the point of saturation. Most of them vomited so freely, that I did not think it necessary to increase that propensity, presuming that a vegetable poison, so comminuted when taken into the stomach, would quickly have its influence elicited and diffused. Some few of them did not vomit at all, yet their recovery was as speedy as in the other cases. The mixture was taken with as much of the carbonic acid gas as they could swallow; many of them expressed expressed that they were greatly relieved by it, and with avidity begged to have more. Now, whether fear, arising from the horrid consideration of being poisoned, and the expectation of this medicine having a certain antidotal power over it, might occasion their anxiety to take so much of it, I will not pretend to determine.

Respecting the probability of a poisonous vegetable being incidentally mixed with grain, or flour, in quantity sufficient to produce such alarming effects, my inquiries have not been satisfactory. I understand that the only noxious substance that is found to grow in abundance, among wheat or oats, is *darnel;* the Lolium *temulentum* of Linnæus, in some counties called *cheal.* The most material information that I have been able to obtain on the effects of darnel, is from the first and second volumes of your Journal, where any of your readers may find the substance of what has been written on the subject by various authors.

If any of your friends should wish to examine the meal in question, I will, on receiving their address, send them a little parcel of it.

These imperfect observations would have been with you by the middle of the month, but I expected to have been able to have said where the oats had grown, and whether the land furnished the darnel in abundance.

I am, Gentlemen,
Your's, &c.

*Stockport, July* 24, 1812. W. LOWE.

